# Relationship between Anxiety and Burnout among Chinese Physicians: A Moderated Mediation Model

**DOI:** 10.1371/journal.pone.0157013

**Published:** 2016-08-01

**Authors:** Jiawei Zhou, Yanjie Yang, Xiaohui Qiu, Xiuxian Yang, Hui Pan, Bo Ban, Zhengxue Qiao, Lin Wang, Wenbo Wang

**Affiliations:** 1 Department of Medical Psychology, Public Health Institute of Harbin Medical University, Harbin, China; 2 Department of Endocrinology, Peking Union Medical College Hospital, Beijing, China; 3 Department of Endocrinology, Affiliated Hospital of Jining Medical College, Jining, China; The University of Queensland, AUSTRALIA

## Abstract

**Objective:**

The main goal of this research was to investigate the complex relationships among coping styles, personality, burnout, and anxiety using a moderated mediation analysis.

**Methods:**

A random cluster sampling procedure was used to select a total of 1274 physicians from two tertiary grade A hospitals in Heilongjiang Province, which is located in northeast China. The Zung Self-Rating Anxiety Scale (SAS), Chinese Maslach Burnout Inventory (CMBI), Chinese version of the EPQ-revised Short Scale, and the Trait Coping Style Questionnaire (TCSQ) were used to gather data. Moderated mediation analysis was used in this study; it was executed using the PROCESS macro so that the mediators and moderator could function together in the same model.

**Results:**

The prevalence of anxiety symptoms among the physicians was 31%, and there were no differences between the sexes. The results showed that positive and negative coping styles partially mediated the association between burnout and anxiety symptoms in physicians. The mediated effect of positive coping styles was moderated by Eysenck’s Psychoticism traits.

**Conclusions:**

Personality traits moderate the strength of the relationships between burnout and anxiety mediated by positive coping styles; however, personality traits do not moderate the strength of the relationships between burnout and anxiety mediated by negative coping styles.

## Introduction

People now expect more from medical services and physicians because of the rapid development of medical treatment. Reform in healthcare is promoted continuously to improve the standard of living; this encourages shifts in medical technology. Physicians, in particular, are at the frontier of the development of medicine. In providing medical services, physicians have not only to attempt to cure patients, but also to confront mental distress, disease, and death. On account of physicians’ long-term exposure to high levels of occupational stress, they tend to be at high risk for mental disorders, such as anxiety and depression [1, 2]; this is also true of physicians in China.

Anxiety is one of the most common psychological conditions. It is a feeling of apprehension, dread, or foreboding accompanied by a variety of autonomic signs and symptoms, in the presence or absence of a stressful situation [3]. Anxiety is a normal reaction to stress, and anxiety can actually be a protective function in some situations. However, when the state of anxiety is severe and continuous, the individual may suffer from an anxiety disorder [4]. Physicians are more prone to anxiety than those in other medical occupations. The prevalence of anxiety among Chinese physicians is markedly higher (25.67%–35.3%) [5] than that in physicians in other countries (2.2%–24%) [6]. Why do Chinese physicians experience so many symptoms of anxiety? First, China has an enormous population base; the ratio of physicians to the general population is 1:735, which is significantly lower than in Western countries (1:280–1:640) [7]. Chinese physicians experience long working hours, tend to work overtime, and have a tremendous workload. Second, deterioration of the physician–patient relationship has become a huge problem in China’s healthcare system [8]. In China, violent incidents between medical staff and patients have increased by 11% annually since 2000. In 2012, at least seven medical staff were killed in hospitals. This is nearly one-half of the sum of deaths in the previous 9 years [9]. Medical conflict has caused countless violent incidents, and has made the relationships between physicians and patients very sensitive. This has become one of the origins of anxiety attacks amongst Chinese physicians.

A recent study suggested that the anxiety state in physicians can adversely affect their health and induce changes in the immune system [10]. Leander et al. [11] reported that anxiety has a large influence on respiratory symptoms. In addition, if a physician has been in a state of anxiety for a long time, it will not only endanger their psychological well-being, but also affect the quality of medical services, the safety of their patients, and it may even affect the stability, unity, and harmonious development of the families of patients and the safety of society. Therefore, for patients, physicians, and society, it is necessary to conduct research on factors that affect anxiety in physicians so that methods can be developed to reduce their anxiety state.

Burnout is a common syndrome for those in many service occupations, such as nurses, physicians, social workers, and teachers. Job burnout is a special type of work-related stress, which can cause a state of physical, emotional or mental exhaustion combined with doubts about competence and the value of work. [12] In Maslach’s model, burnout is the result of chronic stress and includes three key components: emotional exhaustion, cynicism or depersonalization, and reduced feelings of personal accomplishment [13]. For physicians, burnout may affect not only their attitude towards patients and patient safety, but also their own health [14]. Job burnout differs from anxiety because job burnout originates from an accumulation of work-related stressors [15]. Recent research suggests that job burnout is one of the risk factors for anxiety and can predict anxiety [16],[17].

To date, the effect of burnout on anxiety symptoms has been tested, but few studies have addressed the potential mechanism underlying the relationship between burnout and anxiety is still not clear, but mediators may be involved. Indeed, coping style is a common mediator [18],[19]. When people are experiencing problematic and traumatic life events, people will often react with cognitive, affective, or behavioral response strategies, i.e. the coping style [20], which is also considered to be part of the potential mechanism of the relationship between burnout and anxiety in this study. Jiang [21] has proposed two kinds of coping style: positive coping and negative coping, which are the main components of ego defense mechanisms, coping behavior, and cognition. Positive coping refers to problem-solving behavior and positive appraisal. Negative coping refers to more emotion-focused and maladaptive coping mechanisms, or not coping [22]. Kobasa [23] has shown that positive coping strategies can facilitate solving problems more effectively and help individuals to avoid excessive negative emotions. A recent study indicated that there is a significant positive correlation between burnout and negative coping [24]. In addition, coping style could be a predictive factor for mental health; a negative coping style is usually associated with a high level of anxiety [25]. Moreover, Folkman and Lazarus [19] reported that coping mediates emotions in stressful encounters; however, burnout was a result of chronic stress. Therefore, coping style might mediate the association between burnout and anxiety. Nevertheless, whether or not coping style mediates the relationship between burnout and anxiety symptoms in physicians has not been investigated.

Recently, there has been a growing interest in investigating personality factors associated with mental health. Personality traits and anxiety disorders are strongly connected. Suls et al. [26] found that individuals who have a high neuroticism dimension tend to have more anxiety attacks. Such individuals are easily incapacitated by stressful events. Vollrath et al. [27] indicated that individuals with a high level of introversion are vulnerable to stress, which leads to anxiety. Anxiety in physicians can cause a lack of proficiency and other dangerous effects that threaten medicine clinician’s effectiveness. Personality traits are stable psychological structures, and therefore can be used as moderating variables for research purposes. In the present study, we investigated the personality traits that may moderate the association between coping style and anxiety.

Although the influence of burnout on anxiety symptoms has been tested, no studies have explored the mechanism underlying the relationship between burnout and anxiety symptoms. To explore the mechanism between burnout and anxiety symptoms, it is important to understand the roles of coping styles and personality traits in this relationship.

**Fig 1:**
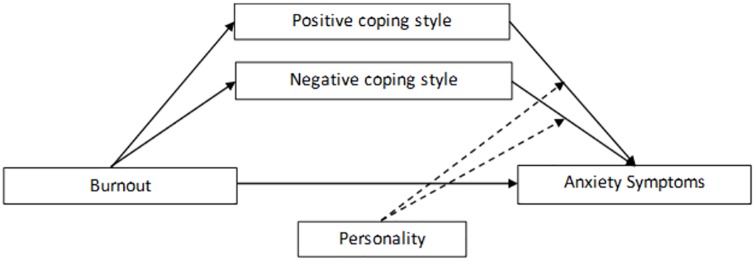
The moderated mediation model applied in this study. The influence of burnout on anxiety symptoms. The effect of burnout is mediated by coping styles. Personality traits further modify the coping process.

In the present study, we determined the prevalence of anxiety symptoms in Chinese physicians and evaluated a moderated mediation model among burnout, coping styles, personality traits, and anxiety symptoms in physicians. Based on the above evidence, we initiated the present study with the following hypotheses: a) the relationship between burnout and anxiety among physicians is mediated, at least in part, by physicians’ coping styles; and b) personality characteristics serve as a moderator between coping and anxiety in physicians. The potential mechanism underlying the relationship between anxiety and burnout involves coping styles mediating the association between job burnout and anxiety symptoms and personality moderating the strength of this association. This research was designed to investigate the potential mechanism involved with burnout and anxiety symptoms to provide evidence to improve the mental status of physicians. The proposed moderated mediation model in the present study is depicted by Fig 1. The effect of burnout is mediated by coping styles. Personality traits further modify the coping process.

## Materials and Methods

### Ethics statement

This study was approved by the Ethics Committee of Harbin Medical University. All participants gave written consent after being informed of the study procedure. We explained the properties, uses, benefits, and adverse effects of the research, and participation was voluntary.

### Participants and procedure

This cross-sectional survey was performed in Daqing city, Heilongjiang Province, which is located in northeast China. There are five “tertiary grade A hospitals” in Daqing City. According to "the hospital classification system" of the Ministry of Health of People's Republic of China, all hospitals in China are classified into primary, secondary, and tertiary hospitals, based on their functions in providing medical care, medical education, and conducting medical research. A tertiary hospital must have a bed number exceeding 500 and provide comprehensive and specialized medical care with a high level of medical education and research functions. Tertiary hospitals are further classified into three subgroups: Grade A, Grade B, and Grade C due to their service levels, medical technology, medical equipment, and management and medical quality. “Tertiary grade A hospital” represents the highest level of medical service in China. The five “tertiary grade A hospitals” were labeled from 1 to 5. With random number table, the researchers choose two hospitals. Cluster sampling procedure was used to recruit a total of 1274 physicians from two tertiary grade A hospitals in Daqing city. One hospital is the Fifth Affiliated Hospital of Harbin Medical University; the other is Daqing Oilfield General Hospital. Here the term “physician” in this manuscript includes medical practitioners from different clinical sections such as Surgery, Cardiology, Pediatrics, etc. Doctors in training had been excluded from this research project. Each of the physicians was provided with a self-administered questionnaire, and 1130 of them returned the questionnaires (response rate = 88.7%). After exclusion of invalid or missing data, 1129 questionnaires (433 physicians from Fifth Affiliated Hospital of Harbin Medical University and 696 physicians from Daqing Oilfield General Hospital.) were valid (88.6%), and these physicians served as our study participants.

### Anxiety symptoms

Anxiety-related symptoms in the physicians were measured by the Zung Self-rating Anxiety Scale (SAS) [28], which was developed in 1971 to assess the severity of anxiety symptoms. The SAS questionnaire includes 20 items, with each item scored on a 4-point scale (1, never or rarely; 2, some of the time; 3, frequently; and 4, most of the time). The minimum raw score is 20, and the maximum raw score is 80; the raw score is multiplied by 1.25, then the integer part is retained in order to generate the index score (range, 25–100). A high score indicates a high level of anxiety. We defined anxiety symptoms to be indicated by a total index score ≥50, according to the Chinese norm [29]. The Chinese version of the questionnaire has been widely used in Chinese populations and has good validity and reliability (Cronbach’s alpha = 0.85) [29].

### Job burnout

The 15-item Chinese Maslach Burnout Inventory (CMBI), as revised by Li et al. [30], was applied to measure burnout in physicians. The inventory includes three dimensions of burnout (emotional exhaustion, depersonalization, and reduced personal accomplishment). Each dimension includes five items, and each item is scored from 1 (never) to 7 (every day). Each item of reduced personal accomplishment is reverse-scored. The cut-off scores for the three dimensions were >25, 11, and 16, respectively. Burnout was divided into four grades, according to the scores on the three dimensions [31]. They are no burnout (scores on all the three dimensions are below the cut-off values), slight burnout (scores on any one of the three dimensions is higher than or equal the cut-off values), moderate burnout (scores on any two of the three dimensions is higher than or equal to the cut-off values), and severe burnout (scores on all the three dimensions are higher than or equal to cut-off). The CMBI has high reliability and validity (Cronbach’s alpha = 0.816), and is suitable for the Chinese cultural background [32, 33].

### Coping style

We used the Trait Coping Style Questionnaire (TCSQ) to measure coping style. It includes 20 items that contain two dimensions of coping (positive and negative coping). Each dimension consists of 10 items, and each item is scored on a 5-point scale, where 1 means ‘certainly not’ and 5 means ‘certainly.’ A higher score on one dimension indicates that the individual is more likely to utilize this type of coping style. The questionnaire is highly reliable and suitable for a Chinese population. The Cronbach’s alpha values for the two independent dimensions of coping were 0.70 and 0.69, respectively [34].

### Personality

Personality was measured using the Chinese version of the Eysenck Personality Questionnaire-revised Short Scale (EPQ-RSC), as translated by Qian et al. [35]. The scale consists of four subscales (neuroticism [N], extraversion [E], psychoticism [P], and lie [L]), and each subscale has 12 items. The scale is suitable for the Chinese population and has good validity and reliability; the internal reliability of each subscale has been measured by Cronbach’s alpha (range, 0.74–0.78; p = 0.6).

### Demographic characteristics

Data on age, sex, marital status, education, day versus night shift, and professional qualifications were obtained in this study. Age was classified as: ≤30 years of age, 31–40 years of age, and ≥41 years of age. Marital status was classified as single, married, or divorced/widowed. The level of education was categorized as junior college or lower, college, and postgraduate or above. Professional qualifications were categorized as primary title, intermediate title, senior vice title, senior title, and none. The effective responses in the present study were obtained from 1274 physicians (471 males [41.82%] and 655 females [58.17%]).

In this study, the four levels of burnout (‘no burnout,’ ‘mild burnout,’ ‘moderate burnout,’ and ‘severe burnout’) were considered to be independent variables. The dependent variable was anxiety. The mediating variables were two dimensions of coping style (positive and negative). The personality traits were considered to be moderating variables.

### Statistical analyses

#### Preliminary analyses

Descriptive analysis, one-way analysis of variance (ANOVA), and an independent samples t-test were used to describe and compare the demographic data (age, marital status, sex, education level, night shift, and professional qualifications) and the distribution of anxiety symptoms. The correlations between the study variables (burnout, coping style, personality traits, and anxiety) were examined using Spearman correlation coefficients. Correlational analyses of the seven variables (anxiety symptoms, burnout level, positive coping, negative coping, extraversion, neuroticism, and psychoticism) were performed using SPSS 21.0 for Windows. Statistical significance was defined as a two-tailed p-value of <0.05.

#### Mediation and moderation analyses

In the mediating and moderating analysis, sex, age, education, and job title were treated as concomitant variables in the regression equations. We examined the mediation effect first. Mediation analysis is used to identify and explicate the relationship between the dependent variable Y and an independent variable X, which may be affected via the interaction of a third variable W. Here, W is a mediating variable, and it represents a mechanism through which X affects Y. In the current study, for example, "burnout" impacts "coping"; with "coping" acting as a mediator variable, which further affects the "anxiety."

Secondly, we conducted the hierarchical regression analysis to test the moderated mediation effects. Each dimension of coping style and each dimension in the personality inventory of Eysenck was created by centering meaning, and a composite variable was created for each interaction. The variables were centered to avoid issues of multi-collinearity [36, 37]. In the first regression, anxiety was regressed on burnout and personality traits. The coefficient for burnout was significant. In the second regression, coping was regressed on burnout and personality. The coefficient for burnout was significant here too. In the third regression, anxiety was regressed on all predictor variables (burnout, personality traits, and coping style), and the coefficient for coping style was significant. In the last regression, anxiety was regressed on burnout, personality traits, coping style and the interaction between coping style and personality traits. If the coefficient for the interaction between coping style and personality traits was significant, moderated mediation has identified [38].

After the criteria for testing moderated mediation were fulfilled, the MODMED 2.0 macro for SPSS, as offered by Hayes [38] was employed to further explore the nature of the moderated indirect effects. PROCESS was operated using one independent variable (burnout level), two mediators (positive and negative coping), three moderators (extraversion, neuroticism, and psychoticism), and one dependent variable (anxiety symptoms). We used 5,000 bootstrap samples in the present study and determined the mediating effect of the 95% confidence interval. In order to better display the moderation effect, we followed Aiken and West’s [36] procedures and the conditional indirect effects were examined at one standard deviation (SD) above the mean, at the mean, and at one SD below the mean for the personality values used as the moderator variable of interest. This analysis was to determine if the slopes of the regression equations for high and low values of the interaction differed from zero.

## Results

### Socio-demographic data and anxiety symptoms

The characteristics of the participants and the distribution of anxiety symptoms are shown in Table 1. We collected data from 475 male (41.82%) and 654 female physicians (58.17%). The average age of the study population was 38.04 ± 7.74 years (mean ± SD). The average score for anxiety symptoms of the participants in this study was 44.88 ± 13.20. The prevalence of anxiety symptoms was 31% and there were no significant differences in anxiety symptoms associated with sex and marital status. Mean comparison showed there were significant associations between anxiety symptoms and age (F = 11.156, P = 0.000), education (F = 4.096, P = 0.017), night shift (T = 5.463, P = 0.048), professional qualifications (F = 8.695, P = 0.000). There is a significant difference of anxiety among people in varied aged groups, different educational background, night shift and the professional levels.

**Table 1:** Participants’ characteristics and the distribution of anxiety symptoms.

	Groups	N(%)	Anxiety symptoms (M±SD)	F/t	p
Sex				0.615	.069
	Male	475(41.82%)	45.17(13.60)		
	Female	654(58.17%)	44.68(12.94)		
Age (years)				11.156	.000
	≤30	168	44.73(13.06)		
	31–40	560	46.62(13.51)		
	>40	394	42.55(12.53)		
Marital status				1.407	.245
	Single	157(14.12%)	45.92(13.83)		
	Married/cohabiting	934(50%)	44.62(13.17)		
	Divorced/separated/widowed	38(3.3%)	47.50(11.09)		
Education				4.096	.017
	Junior college	142(12.97%)	44.57(13.56)		
	College	573(50.62%)	45.94(13.68)		
	Postgraduate or above	414(36.41%)	43.52(12.28)		
Night shift				5.463	.048
	Yes	652(57.82%)	46.73(13.43)		
	No	477(42.18%)	42.35(12.47)		
Professional qualifications				8.695	.000
	Primary title	229(20.34%)	47.16(14.71)		
	Intermediate title	362(32.06%)	46.59(13.24)		
	Senior vice title	264(23.27%)	44.04(12.07)		
	Senior title	184(16.34%)	40.68(12.12)		
	None	90(8.00%)	43.29(12.06)		

### Preliminary analyses

Table 2 provides the means, SD, and correlations between the seven variables. These results indicate that anxiety symptoms are positively associated with the level of burnout and negative coping. Positive coping was inversely correlated with anxiety symptoms. The level of burnout was positively associated with negative coping and the N dimension in the personality inventory of Eysenck [35], and negatively related to positive coping. The N dimension in the personality inventory of Eysenck [35] was inversely related to positive coping and positively correlated with negative coping. The p values for the associations between the variables were significant, but it should be noted that the correlation coefficients were very small. Thus, the independent variable and moderators have relative independence, which may allow subsequent moderation analysis.

**Table 2:** Correlations between anxiety symptoms, burnout, coping styles, and personality traits (N = 1129).

Variables	1	2	3	4	5	6	7
1. Anxiety symptoms	1.00						
2. Burnout level	.45**	1.00					
3. Positive coping	–.36**	–.33**	1.00				
4. Negative coping	.46**	.34**	–.32**	1.00			
5. Extraversion	.06*	–.01	.06	–.01	1.00		
6. Neuroticism	.16*	.10**	–.08*	.12**	–.34**	1.00	
7. Psychoticism	–.06	–.01	.03	–.00	–.43**	.39**	1.00
Mean	44.88	1.04	34.99	27.47	6.40	5.88	5.09
Standard deviation	13.20	.92	7.15	7.83	3.23	3.68	4.05

Note:

** p < 0.01,

* p < 0.05.

### Mediation analyses

The results of the mediation analysis showed that the level of burnout was inversely related to positive coping, and was significantly associated with negative coping. Positive coping had a negative effect on anxiety symptoms, and negative coping had a positive effect on anxiety symptoms (all p values <0.01 [Table 3]). The level of burnout was also significantly correlated with anxiety symptoms in both models (all p values <0.01 [Table 3]). These results indicated that the relationship between burnout level and anxiety symptoms was partially mediated by positive and negative coping, respectively. Table 4 shows the bootstrapped 95% confidence interval (CI), which confirmed that the indirect effects of each type of coping style in the relationship between burnout level and anxiety symptoms were significant.

**Table 3:** Mediation analysis (N = 1129).

Variables	B	SE	t	p
Burnout level→Positive coping	–2.53	.22	–11.56	<.001
Burnout level→Negative coping	2.89	.24	12.11	<.001
Positive coping→Anxiety symptoms	–.42	.05	–8.33	<.001
Negative coping→Anxiety symptoms	.58	.04	13.04	<.001
Burnout level→Anxiety symptoms	6.533	.38	17.24	<.001

**Table 4:** Bootstrap results for indirect effect (N = 1129).

	Bootstrap results for indirect effect			
Variables	Effect	SE	LL 95% CI	UL 95% CI
Positive coping	1.0617	.1784	.7315	1.4319
Negative coping	1.6711	.1955	1.3141	2.0791

### Moderated mediation analyses

When moderated mediation analysis was performed, positive and negative coping appeared to be significant mediators. Table 5 shows the results of the moderated mediation analysis when treating positive coping as the mediator, and when the E, N, and P dimensions in the personality inventory of Eysenck [35] were used as moderators in the relationship between burnout level and anxiety symptoms. Table 6 presents the results of the moderated mediation analysis when negative coping was entered as the mediator, and when the E, N, and P dimensions in the personality inventory of Eysenck [35] were entered as moderators in the association between burnout level and anxiety symptoms.

**Table 5:** Moderated mediation analysis when treating positive coping as a mediator (N = 1129).

	Outcome variable: anxiety symptoms			
Variables	B	SE	t	p
Burnout	5.25	.39	13.64	.000
E dimension	.45	.13	3.62	.000
N dimension	.67	.11	6.07	.000
P dimension	–.18	.11	–1.72	.085
Positive coping (POC)	–.34	.09	–3.85	.000
POC×E	.04	.02	2.65	.008
POC×N	.01	.01	1.05	.295
POC×P	–.01	.02	–.81	.417
R^2^	.314			

**Table 6:** Moderated mediation analysis when treating negative coping as a mediator (N = 1129).

	Outcome variable: anxiety symptoms			
Variables	B	SE	t	p
Burnout	4.70	.37	12.59	.000
E dimension	.35	.12	2.99	.003
N dimension	.59	.12	5.02	.000
P dimension	–.24	.10	–2.28	.023
Negative coping (NEC)	.55	.08	6.76	.000
NEC×E	–.01	.01	–.40	.692
NEC×N	.00	.01	–.06	.952
NEC×P	.02	.01	1.14	.255
R^2^	.361			

Because the results of mediation have already been reported in the previous paragraph, we reported the interaction effects between the variables included in the regression model directly. The results suggested that the interaction between positive coping and the E dimension (POC×E [Table 5]) was significant for anxiety symptoms (p = 0.008). Thus, the effect of positive coping on anxiety symptoms was moderated by the E dimension in the personality inventory of Eysenck [35]; however, the interactions between negative coping and the dimensions in the personality inventory of Eysenck [35] (NEC×E, NEC×N, and NEC×P [Table 6]) were not significant.

**Fig 2:**
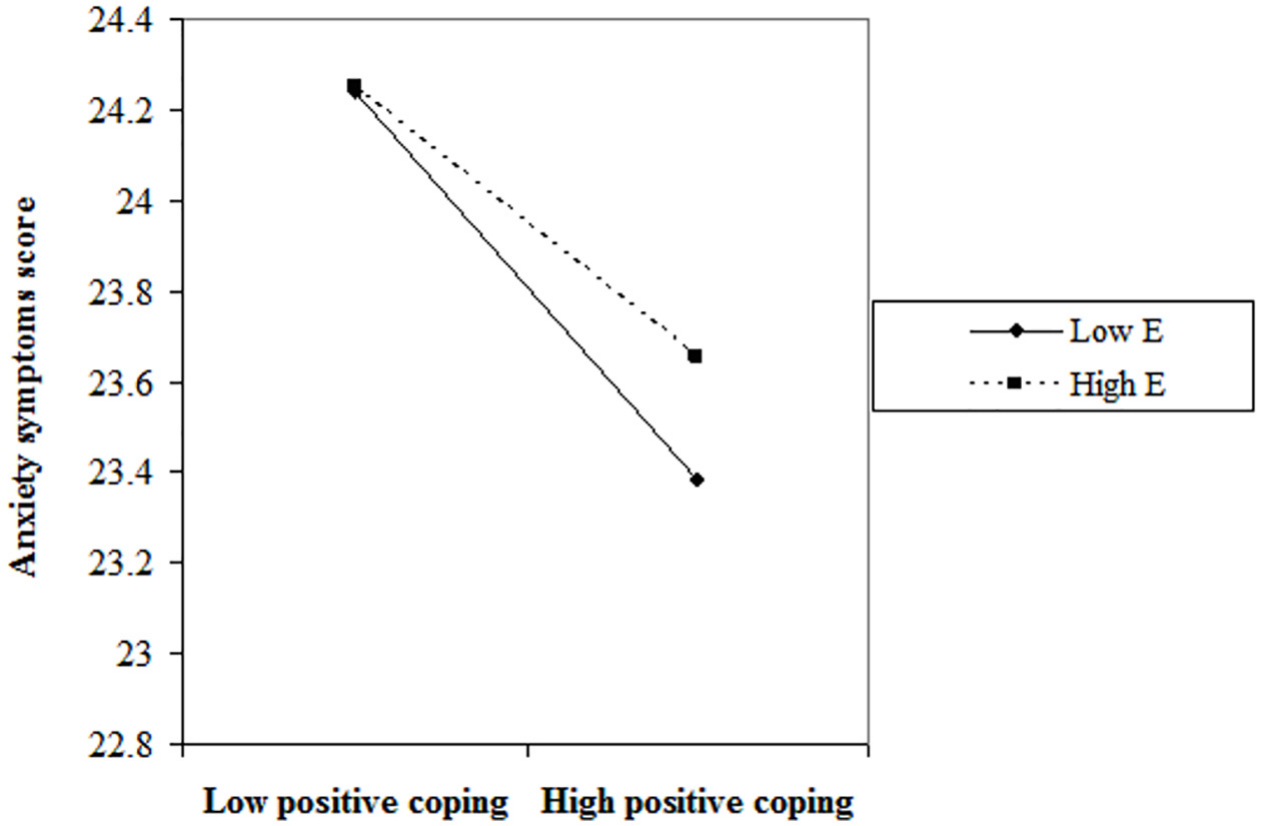
Moderating effect. E dimension moderates the relationship between positive coping and anxiety symptoms among Chinese physicians.

The effects of positive coping on anxiety symptoms were examined by a simple main effects analyses at 1 SD above and below the mean of the E dimension in the personality inventory of Eysenck [35]. At the high level (mean +1SD) of the E dimension, the main effect of positive coping was significant. Thus, the higher level of positive coping was related to a lower level of anxiety symptoms (β = −.149, p <0.001). Although higher levels of positive coping were significantly associated with lower levels of anxiety symptoms at low levels (mean –1SD) of the E dimension, when compared with the high level of the E dimension from the chart, the low level of the E dimension gave a steeper line. When positive coping increased, the anxiety level decreased further (β = −.575, p <0.001). Fig 2 depicts positive coping moderated by the E dimension in the personality inventory of the Eysenck interaction.

**Fig 3:**
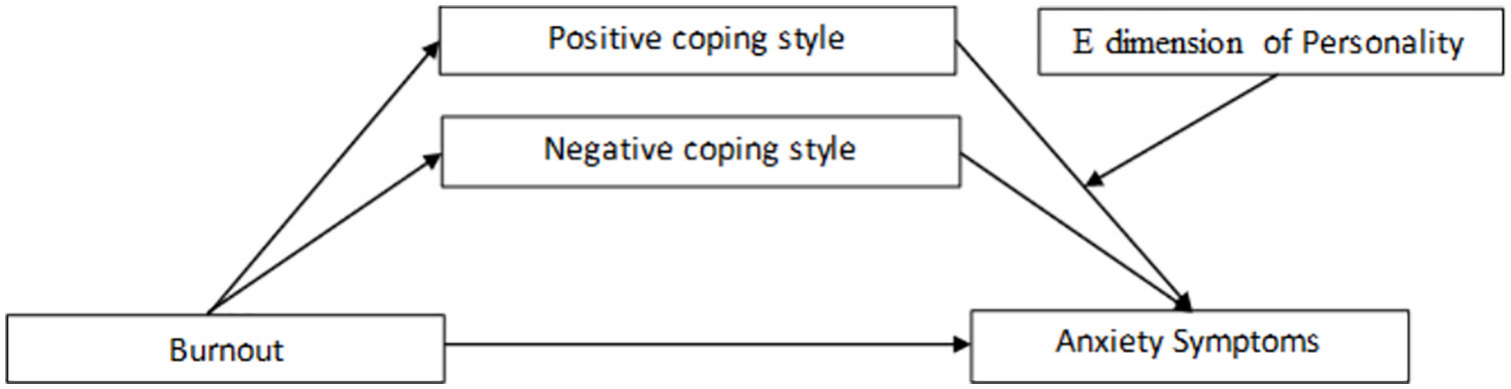
The final moderated mediation model. The link associating the burnout and positive coping with anxiety is moderated by personality traits.

To evaluate the conditional indirect effects of the level of burnout on anxiety symptoms via positive coping, as a function of different ranges of the E dimension, we used the bootstrap method for analysis. Indirect effects at three levels of the E dimension (1 SD above the mean, at the mean, and 1 SD below the mean) were examined by using the 95% CIs of the bootstrap method. As shown in Table 7, the conditional indirect effect on anxiety symptoms arose from burnout via positive coping. This effect changed according to the range of the E dimension and was weakest at 1 SD below the mean of the E dimension. These results indicate that the more burnout a physician has, the more susceptible they are to the development of anxiety symptoms. Physicians with lower levels of the E dimension who use positive coping can achieve better protective effects than those with higher levels on the E dimension. The final moderated mediation model is displayed in Fig 3.

**Table 7:** Conditional indirect effect at specific levels of the moderator when treating positive coping as a mediator (N = 1129).

Moderator: level of E	Indirect effect	SE	LL 95% CI	UL 95% CI
1 SD above the mean	1.2815	.2267	.8826	1.7602
Mean	1.0856	.1781	.7696	1.4867
1 SD below the mean	.8897	.2106	.4981	1.3344

## Discussion

This study investigated the prevalence of anxiety symptoms among physicians in a region of China and explored a model of the relationship between job burnout, personality traits, coping style, and anxiety symptoms via a moderated mediation analysis. The prevalence of anxiety symptoms in the physicians was 31%; there were no differences associated with sex. This rate was higher than the prevalence in physicians in Amsterdam, the Netherlands (24%) [39], more than double the prevalence in medical university students and middle school teachers in China [40], and also higher than the prevalence in Chinese physicians who worked about 17 years ago (21.05%) [41]. Therefore, it is necessary to explore the factors that affect anxiety symptoms in physicians.

Our results showed that there is a strong positive correlation between job burnout and anxiety symptoms in physicians; this is generally consistent with the results of previous studies [42–44]. The Chinese Maslach Burnout Inventory (CMBI) is a modified version of Maslach Burnout Inventory (MBI). It is suitable for the Chinese cultural background and language habits. The CMBI has high reliability and validity (Cronbach’s alpha = 0.816). Therefore, CMBI was applied to measure burnout in physicians in the present study. Burnout is a problem that is specific to the work context, and is characterized as emotional exhaustion, cynicism, and reduced professional efficacy [13]. In China, job burnout has become common among physicians because of the long working hours, heavy workload, and imbalance of effort/reward [45]. Physicians with a high level of job burnout may feel emotionally overloaded with work, have a negative working attitude towards patients, or even feel a lack of productivity and achievement at work. Physicians who experience burnout syndrome appear to be more prone to developing anxiety. Thus, attention should be given to job burnout to reduce symptoms of anxiety in Chinese physicians. Health managers should not only pay more attention to job burnout syndrome in the workplace among Chinese physicians, but also take measures to reduce burnout syndrome, such as reducing working hours, ensuring a safe working environment, and increasing salaries.

Greater attention should be paid to methods to reduce the anxiety symptoms of Chinese physicians. In this study, we attempted to demonstrate the potential relationship between job burnout and anxiety symptoms. We anticipate that our results will contribute to identifying effective interventions to reduce anxiety in physicians.

Job burnout not only had a direct effect on anxiety symptoms in physicians, but it also had an indirect influence on anxiety symptoms, via mediation by coping styles. The study also examined the relationships between coping styles and anxiety symptoms: a positive coping style was negatively related to anxiety symptoms; however, a negative coping style had a strong positive relationship with anxiety symptoms. Furthermore, the results showed that the two coping styles partially mediated the association between job burnout and anxiety symptoms. These results are consistent with those of previous studies showing that coping styles are frequent mediators of many symptoms, such as anxiety and depression [46, 47]; however, different coping styles had different roles in mediation. The present study showed that a positive coping style partially mediated the association between job burnout and anxiety, as a protective factor. The physicians who had a low level of job burnout were more likely to use a positive coping style to solve problems, which in turn led to a reduction in anxiety symptoms. The results also showed that the relationship between job burnout and anxiety was partially mediated via a negative coping style, as a risk factor. Physicians who had a high level of job burnout were more likely to use a negative coping style to solve problems, which in turn led to an increase in anxiety symptoms. Based on the study by Jiang [48], use of a positive coping style can alleviate psychosomatic symptoms; however, an inappropriate coping style will increase the stress response. In the present study, coping styles were verified to be mediators between burnout and anxiety. Therefore, for physicians with burnout, it is important to encourage a positive coping style rather than a negative coping style, in order to reduce symptoms of anxiety.

In particular, our integrated moderated mediation analyses demonstrated general support for our second hypothesis (b). The study showed that personality traits moderate the strength of the relationships between burnout and anxiety mediated by a positive coping style. However, personality traits did not moderate the strength of the relationships between burnout and anxiety mediated by a negative coping style. We demonstrated that a positive coping style partially mediated the association between job burnout and anxiety symptoms, and the strength of the mediating effect of a positive coping style on the relationship between job burnout and anxiety symptoms was moderated by an extraverted personality. Furthermore, our results showed that a low level of extraversion attenuated the relationships among job burnout, positive coping style, and anxiety symptoms through a link between a positive coping style and anxiety symptoms. Adopting a positive coping style can reduce anxiety symptoms in physicians who have burnout syndrome and, in physicians with a low score for extraversion, use of positive coping strategies may be effective in reducing anxiety symptoms. Our findings may increase understanding of the interactive mechanisms between job burnout and anxiety symptoms, and specifically among Chinese physicians. To reduce the negative consequences of burnout, physicians should change their cognitive strategies, utilizing a positive coping style. Moreover, we should adopt different intervention models for the different traits of individual personalities, in order to reduce anxiety symptoms.

This study has several limitations. The first limitation of our study was the cross-sectional design, so the results should be interpreted cautiously. Therefore, future research should conduct longitudinal or experimental studies to confirm the conclusions of this study. Secondly, self-reported measures were used in our study and the participant response bias is therefore unavoidable. Thirdly, the sample in this study was only randomly selected from tertiary grade A, comprehensive hospitals of Heilongjiang Province, which can hardly be used to generalize our findings to all healthcare professionals in China. Other types of hospitals physicians will be investigated in our future studies.

## Conclusion

This is the first investigation of an association between job burnout and anxiety symptoms among Chinese physicians using the moderated mediation model. The prevalence of anxiety symptoms among physicians was 31% in this study, and there was no difference associated with sex. Coping styles partially mediated the association between job burnout and anxiety symptoms. Finally, possession of an extraverted personality moderated the strength of the relationship between job burnout and anxiety symptoms mediated by a positive coping style; the mediated relationship was stronger in those with a low extraversion score than in those with a high extraversion score.

## Supporting Information

S1 Dataset Dataset of the subsamples (SAV). (SAV)
